# Notes and News

**Published:** 1902-01-25

**Authors:** 


### NOTES AND NEWS.

AUSTRALIA is following the lead of America in bringing the law to bear upon the habit of spitting in public places and causing a danger to health.

A CHILDREN'S ward has been opened at the Mercers' Hospital, Dublin. It was previously used for adult patients, but the need of a separate ward for children being recognised, the alteration was made.

A NEW ward has been opened at the Children's Hospital, Pendlebury, Manchester. The new ward was, up to the time of the erection of a nurses' home, used to accommodate the nurses. The ward will accommodate twenty-six cots, and will be called the Queen Victoria Ward.

A CONSUMPTION sanatorium is to be established in the Blue Mountains in New South Wales. Only a few patients will be received at first, and apparently the institution is to be regarded somewhat as an experiment. The salary of £300 for the medical director is intended to tempt someone who, slightly tuberculous himself, will reap a benefit beyond that which the salary will offer. But experience shows that where services are demanded, a strict business basis is best in the interests of everyone. The sanatorium will be in connection with the Queen Victoria Home for Consumptives in Sydney.

A CAUSE of death is met with in the United States which fortunately does not appear in English returns, namely, lynching. According to the Chicago *Tribune* the lynchings which took place last year in the States outnumbered the official executions. During the year 1901 there were 118 legal executions, 71 of those executed being negroes and 47 whites. But there were 135 lynchings, an increase of 20 over the year before, and of the victims 107 were negroes, one was an Indian, and one a Chinaman, and only 26 were whites. A very important point in the increase of lynchings (14 as compared with 8 in 1900) in the more Northern States, six of these cases having taken place in California and four in Montana. It would seem that life is held somewhat cheap in many parts of the States. During the year 1901 there were 7,245 suicides, and 7,852 murders, the latter being in striking contrast to the 118 official executions.

LADY WANTAGE has given £1,000 to the Charing Cross Hospital in connection with the forthcoming festival dinner, in memory of Lord Wantage, late President.

THE completion of the New General Hospital, Birmingham, was celebrated by a banquet given by Sir John Holder to the general committee, the staff of the hospital, and personal friends.

PHYSICAL training is everywhere receiving an impetus. In the opening of classes at the Alexandra Palace, by the Princess Louise, the urban suburban population will receive added encouragement and opportunity.

THE Congress of the Sanitary Institute will be held in September at Manchester this year, the presidents of the various sections being Sir James Crichton-Browne, Sir Alexander Binnie, and Prof. Sheridan Delpéine.

To those who are interested in the refugee camps in South Africa with the attendant problems on hygiene and sanitation, the Blue Book just issued entitled "Further Papers Relating to the Working of the Refuge Camps in South Africa" will be most acceptable.

THE Dorsetshire County Asylum, which for some years has received private patients, is now making preparations for a more extensive adoption of the system. Plans have been submitted and approved for the building of a house for one hundred private patients, at a cost of £40,000.

MR. MYERS, of Birmingham, has founded a travelling studentship in the University of Birmingham in memory of his son, D. Walter Myers, who died in the service of the Liverpool School of Tropical Medicine. The student may pursue studies in pathology and clinical medicine, and the value of the studentship is £150 for one year, which may be extended with reduced remuneration.

IN a report dealing with the outbreak of small-pox in London the Public Health Committee of the London County Council recommend that the Council should make an order under Section 56 of the Public Health (London) Act, 1891, temporarily rendering chicken-pox a notifiable disease. The report states that seven of the metropolitan borough councils—Hampstead, St. Pancras, Hackney, Holborn, Bethnal Green, Greenwich, and Paddington—have already taken steps to render notification of chicken-pox obligatory in their districts, and to this list we may now add the City of Westminster.

PERHAPS the most convincing proofs in favour of vaccination published in the recent report of the Statistical Committee of the Asylums Board, is that which deals with the employees, whom everyone will admit run great and reiterated risks in following their occupation. During the last ten years, 1,282 persons have been employed in the ambulance service of the Board. They are subjected to adequate re-vaccination. Of the number quoted, two deaths from small-pox occurred. One of these had escaped re-vaccination, and another had been unsuccessfully re-vaccinated. Two only besides these contracted the disease at all.

